# Changes in pro-inflammatory markers and leucine concentrations in response to Nordic Walking training combined with vitamin D supplementation in elderly women

**DOI:** 10.1007/s10522-017-9694-8

**Published:** 2017-03-18

**Authors:** A. Gmiat, J. Mieszkowski, K. Prusik, K. Prusik, J. Kortas, A. Kochanowicz, A. Radulska, M. Lipiński, M. Tomczyk, J. Jaworska, J. Antosiewicz, E. Ziemann

**Affiliations:** 1 Department of Physiology and Pharmacology, Gdansk University of Physical Education and Sport, Kazimierza Górskiego 1, 80-336 Gdańsk, Poland; 2 Department of Anatomy and Biomechanics, Institute of Physical Education, Kazimierz Wielki University, Bydgoszcz, Poland; 3 Department of Recreation and Qualify Tourism, Gdansk University of Physical Education and Sport, Gdańsk, Poland; 4 Department of Gymnastics and Dance, Gdansk University of Physical Education and Sport, Gdańsk, Poland; 5 Department of Biochemistry, Medical University of Gdańsk, Gdańsk, Poland; 6 Department of Pharmaceutical Biochemistry, Medical University of Gdańsk, Gdańsk, Poland; 7 Department of Biochemistry, Gdansk University of Physical Education and Sport, Gdańsk, Poland; 8 Department of Bioenergetics and Physiology of Exercise, Medical University, Gdańsk, Poland

**Keywords:** Aging, High-Mobility Group Box 1 (HMGB1), Branched chain amino acids

## Abstract

Mechanisms underpinning age-related decreases in muscle strength and muscle mass relate to chronic inflammation. Physical activity induces an anti-inflammatory effect, but it is modulated by additional factors. We hypothesized that vitamin D, which has also anti-inflammatory activity will modify adaptation to exercise and reduce inflammation in elderly women. Twenty-seven women aged 67 ± 8 years were included and divided into groups with baseline vitamin D concentration more than 20 ng mL^−1^ (MVD) and less than 20 ng mL^−1^ (LVD). Both groups performed 1 h Nordic Walking (NW) training combined with vitamin D supplementation for 12 weeks. Serum concentrations of inflammation markers, branched amino acids, vitamin D, muscle strength and balance were assessed at the baseline and three days after intervention. The training caused the significant decrease in concentration of pro-inflammatory proteins HMGB1 (30 ± 156%; 90% CI) and IL-6 (−10 ± 66%; 90% CI) in MVD group. This effects in group MVD were moderate, indicating vitamin D as one of the modifiers of these exercise-induced changes. Rise of myokine irisin induced by exercise correlated inversely with HMGB1 and the correlation was more pronounced at the baseline as well as after training among MVD participants. Although the intervention caused the leucine level to rise, a comparison of the recorded response between groups and the adjusted effect indicated that the effect was 20% lower in the LVD group. Overall the applied training program was effective in reducing HMGB1 concentration. This drop was accompanied by the rise of myokine irisin and better uptake of leucine among women with higher baseline vitamin D.

## Introduction

Aging is associated with a number of structural and functional changes that are conducive to a progressive increase in body fat and a corresponding decline in muscle mass (Lee et al. 2016). Slow and continuous loss of muscle mass and muscle strength that progresses with aging is defined as “sarcopenia” (Santilli et al. 2014). It represents an important public health problem closely linked with a risk of falls and injuries, enhanced functional limitations and disability, which restrict one’s independence (Janssen et al. 2002).

Sarcopenia has been widely reported, but the associated physiological mechanisms remain unclear. Muscle loss accompanying aging can be attributed to complex interactions between factors including: muscle protein turnover, rising anabolic resistance and delayed amino acid absorption, endocrine system functioning, alterations of the neuromuscular junction as well as behavior- and disease-related factors (Curcio et al. 2016). Negative balance of muscle protein may result from a reduced rate of muscle protein synthesis and an increased rate of muscle protein breakdown. Amino acids were shown to induce a muscle protein anabolic response conditioned by the availability of branched-chain amino acids (such as leucine, isoleucine, valine) (Walker et al. 2011). Delayed amino acid absorption and anabolic resistance are frequently noted among elderly; thus, the assessment of a plasma/serum free pool of amino acids may characterize the anabolic activity of an aged muscle mass (Burd et al. 2013).

Age-related muscle wasting involves not only tissue loss, but also endocrine and metabolic abnormalities linked with systemic inflammation (Beyer et al. 2012), which the endogenous nuclear High-Mobility Group Box 1 (HMGB1) is involved in (Yang et al. 2015). HMGB1 becomes a pro-inflammatory cytokine, which enhances cell migration, affects cell proliferation and activates the inflammatory condition (Klune et al. 2008; Smolarczyk et al. 2012). An elevated concentration of HMGB1 was observed among diabetic and obese adults (Golbidi et al. 2012) and the protein was also found to positive correlate with the body mass index (Wang et al. 2015).

On the other hand, muscle mass was demonstrated to act as a secretory organ, releasing proteins (myokines) into the bloodstream during contractions (Pedersen 2011). Most of them induce an anti-inflammatory effect, causing reduction of inflammation, associated with aging (Petersen and Pedersen 2005). Two myokines in particular were described in greater detail, interleukin 6 (IL-6) and irisin (Bostrom et al. 2012; Pedersen 2011). IL-6 is a pleiotropic protein that acts as both a pro-inflammatory cytokine and an anti-inflammatory myokine, which grows in response to muscle contraction (Pal et al. 2014). Irisin is a part of the Fibronectin type III domain-containing protein 5 (FNDC5), released from muscles immediately after exercise, converting white adipose tissue into brown adipose tissue and regulating energy expenditure (Bostrom et al. 2012). It was proposed that this molecule plays an important role in various conditions such as inflammation, hippocampal neurogenesis, aging and other metabolic conditions. Recent research, however, shows that the influence of irisin and the effect of its action are conflicting (Panati et al. 2016).

The anti-inflammatory action may be supported by vitamin D3 (25-hydroxyvitamin D (25-OH-D) due to protection against muscle degradation it provides (Girgis et al. 2015). Vitamin D deficiency, however, is a long recognized problem of civilization (Lips 2001). Documented presence of vitamin D receptors (VDR) in almost all tissues and organs has prompted intense research into vitamin D effects on regulatory adaptive changes. A merge of vitamin D and acute exercise has recently been proposed to modify the insulin growth factor system for enriched muscle well-being (Darr et al. 2016). Consequently, vitamin D is considered to be beneficial in treating sarcopenia. Still, literature data report diverse doses of vitamin D supplementation, highlighting a range of responses recorded in response to exercise and supplementation applied together (Rosendahl-Riise et al. 2017).

There is an important correlation between inactivity and loss of muscle mass and strength (Oh et al. 2016). Compelling data support the efficacy of exercise in improving muscle mass and muscle function in aging populations. For example (Bruseghini et al. 2015) observed that 8 weeks of high intensity interval training and isoinertial resistance training induced hypertrophy of the quadriceps muscle. Also aerobic exercise combined with resistance training was shown to have a greater anti-inflammatory effect than each activity separately (Hopps et al. 2011). In addition, endurance exercise was found to induce an increase in the brain-derived neurotrophic factor (BDNF), which is essential not only in supporting brain plasticity, but also in peripheral metabolism (Pedersen et al. 2009). Data published by Wrann et al. reveal that an increase in irisin, in response to exercise, lead to BDNF concentration growth as well as improvement in cognitive functions (Wrann et al. 2013).

Among different forms of training, Nordic Walking (NW) is a simple and safe form of exercise for elderly people. If practiced regularly, specially designed poles used to push against the ground with each stride, activate both lower and upper body, promoting gain in muscle strength (Song et al. 2013). Ossowski et al. (2016) demonstrated that short-term Nordic Walking training induces positive changes in knee muscle strength and functional performance in women with low bone mass.

In light of these developments, the aim of this study was to evaluate the effect of Nordic Walking training under vitamin D supplementation on branched-chain amino acid and selected pro- and anti-inflammatory proteins’ concentrations, muscle strength and balance. According to our knowledge, there has been no study verifying correlations between HMGB1 and myokines.

## Materials and methods

### Ethics statement

This study was officially approved by the Bioethical Committee of Regional Medical Society in Gdańsk, according to the Declaration of Helsinki, under the process number KB-29/14. Before commencing the study, subjects received a verbal description of the experiment and signed an informed consent form of participation. The ethics approval was obtained for a referral of participants to their family physician upon detection of any abnormal pathology results and a review by the study’s medical officer.

### Subjects

A group of 27 elderly women (67 ± 8 years old) took part in the experiment. All participants were recruited from a group of volunteers, who participate in health promotion programs at the Gdansk University of Physical Education and Sport (41 volunteers). Each of the women had to be medically examined before the experiment. They were also asked to provide information regarding prescribed medications. The exclusion criteria for participation included: uncontrolled hypertension (diastolic blood pressure over 100 mmHg), history of cardiac arrhythmia, cardio-respiratory disorders and some orthopedic problems. Additionally, only women attending at least 90% of training units were taken into consideration. As a result 27 women remained in the experiment. It was recommended that participants did not change their lifestyle and diet throughout the experiment. The analysis and training program were completed at the Gdansk University of Physical Education and Sport.

### Experimental design

Body composition, muscle strength and aerobic capacity were determined one week prior to the start of the experiment and after 12 weeks of training. Blood samples were collected at two time points of the experiment: before starting the NW training and three days after the last training session. All participants completed the NW training and during the training period took two randomly chosen doses of vitamin D3 supplements (800 or 4000 IU/day). For the purpose of statistical analysis, subjects were divided into two sub-groups, characterized by different vitamin D baseline blood levels (LVD less than 20 ng mL^−1^ and MVD more than 20 ng mL^−1)^; however, supplementation varied across sub-groups.

### Exercise protocol

The experimental group completed 12 weeks of NW training, which included 35 training units. The same group of research assistants and coaches supervised all training sessions. Participants met three times a week (Monday, Wednesday and Friday), 1 h after eating a light breakfast. Each training session lasted 1 h (10-min warm-up, 40-min NW, and 10-min cool-down) and had a 60–70% intensity of the maximal ability based on a 2,000 m walking Each training unit was recorded using Garmin Forerunner 405 with a built-in GPS. Each participant received a sport-tester type device used for cardiovascular control During the whole training program participating women covered a total distance of 107 km 300 m (Kortas et al. 2015).

### Blood collection

Blood samples were taken from the antecubital vein into vacutainer tubes at baseline and three days directly after the 12-week training program. The blood was collected at rest, fasting, in the morning hours 7:00–8:00 a.m. Immediately following blood collection one portion of the sample (for irisin assessment) was transferred to centrifuge tubes containing aprotinin (catalog no RK-APRO) from Phoenix Pharmaceuticals Inc. The final concentration of aprotinin was 0.6 Trypsin Inhibitor Unit/1 mL of blood. The samples were centrifuged at 2000×*g* for 10 min at 4 °C and stored at −80 °C until later analysis. Quantification of plasma irisin was based on a competitive enzyme immunoassay and the assay kits were purchased from Phoenix Pharmaceuticals Inc (catalog no EK 067-16). The intra-assay coefficients of variability (CVs) and inter-assay CVs reported by the manufacturer were 4–6 and 8%–10%, respectively. Serum IL-6 and IL-10 was determined by enzyme immunoassay methods using commercial kits (R&D Systems, USA). The detection limits for IL-6 and IL-10 were 0.500 and 0.038 pg.mL^−1^, respectively.

Serum HMGB1 was also assessed by enzyme immunoassay method assessed using reagents Cloud Clone Corp. SEA399hu. The average intra-assay CV was <8.0% for all cytokines.

Serum BDNF was also detected using sandwich ELISA according to the manufacturers’ instructions (R&D Systems, USA; DY248). Detection limit for BDNF was 15 pg mL^−1^. Values are expressed as ng mL^−1^. Based on our previous experiences and Maffioletti’s recommendation, a 1 h clotting duration for a correct serum BDNF dosage was applied (Witek et al. 2016).

### Determination of serum vitamin D concentration

Determination of vitamin D metabolite, 25-hydroxy D3 (25OHD3), concentration was carried out on the basis of “Ultra-fast LC/MS/MS Analysis of 25-OH Vitamin D2 and D3 from serum” with modifications procedure using Phenomenex TN-1055 application. A mass spectrometer SHIMADZU LCMS 8050 HPLC system Nexera X2 column, Agilent Eclipse Plus C18 1.8 μm 2.1 × 100 mm was used for the assay. The mobile phase consisted of 0.1% formic acid in water and 0.1% formic acid in methanol containing 5 mm ammonium acetate. Total Flow 0.15 mL/min. The two passages for ionic 25-OH-D3: m/z 401.2 > m/z 383.4 and m/z 401.2 > m/z 365.35 in the presence of a standard tritiated D2 mz 416.4 > m/z 340.3 were used. The mixture of acetonitrile, methanol, and vitamin D2 [H3] was added to a serum. The sample was mixed and centrifuged, the supernatant was transferred to a separate vial for analysis.

### Analysis of amino acid profile

Amino acids (AA) profile (including leucine, isoleucine and valine) was conducted using ion-pair reversed phase high performance liquid chromatography combined with tandem mass spectrometry IP-RP HPLC–MS/MS (TSQ Vantage Thermo Scientific). The procedure was modification of earlier described by our group (Adrych et al. 2010; Swierczynski et al. 2009). Separation of AA was performed on 2.5 µM 5 × 2 mm Synergi hydro RP (Phenomenex) column using 5 mm NFPA (nonafluoropentanoic acid) as an ion-pairing agent in water (phase A) and Acetonitrile with 0.1% FA (formic acid) (phase B). Calibration curve was prepared to cover physiological concentration of AA in human serum. 10 µM 2-Cl-adenosine and 50 µM homoarginine were used as an internal standard (IS). Acetonitrile was used for protein precipitation. Quality Control sample was treated the same way as samples tested. Positive ionization mode was used and AA were detected and identified based on their fragmentation pattern. The most intense product ions were used for quantification.

### Body composition assessment

Body mass (BM) and total body water, skeletal muscle mass and body fat were measured using a multi-frequency impedance plethysmograph body composition analyser (In Body 720, Biospace, Korea). This analyzer separates total body water into intracellular and extracellular water; it also accurately displays skeletal muscle mass separately from soft lean mass. In addition, the body mass index was calculated. Precision of the repeated measurements was expressed as the coefficient of variation, which on average was 0.6% for fat mass percentage (Volgyi et al. 2008). During measurements, participants wore only briefs and remained barefoot.

### Muscle strength assessment

Isometric and isokinetic elbow and knee muscle functions were measured using a Biodex System 4 dynamometer (Biodex Medical Systems, Inc., Shirley, NY, USA). Data collection was performed using a Compaq Desk Pro personal computer and Biodex software following the standard Biodex protocol. After a 20-min standardized warm-up, subjects were positioned in the equipment according to the manufacturer’s manual. Measurements of the peak torque were taken for the flexion (EF) and extension (EE) at the elbow joint in the conditions of a 5-s isometric contraction. All tests were conducted in a sitting position with a trunk and lower limbs stabilized with belts. Each test was conducted in a random order. After receiving explanations, subjects were familiarized with the procedure by performing one set of submaxi- mal contractions. Each participants was familiar with the test procedure the day before commencement of the muscle strenght assessment. Standardized verbal instructions were given to all subjects during the testing procedure. Each of the peak torque measurements for particular joints was made three times with one-minute breaks in between. Flexion and extension of the elbow were tested in a position with the joint supported and flexed at 90° and the glenohumeral joint flexed at 45°. The next analyzed element was the ratio of opposing peak torques for each of the joints. Therefore, the study covered the flexion/extension ratio at the elbow joint (EF/EE) (Bohannon et al. 2011; Erol et al. 2008).

### Body balance assessment

Body balance was measured in the morning in a quiet indoor laboratory on an AccuGait force platform, recording displacement of the center of pressure (COP) using the AMTI software. Each trail lasted 30 s. Measurements of static postural control in the upright position on both legs with eyes open (EO) and closed (EC) was conducted with a frequency sampling of 100 Hz and low pass filtered at 5 Hz, using a rectangular filter in the frequency domain. Same conditions were fulfilled for postural control on one leg (SR: single right, SL: single left). All subjects were monitored by an observer for safety. During EO and EC trails participants’ feet were placed parallel and at hip width. In each conditions of the test measurements were repeated tree times, and for the analyzes the average value of the tests was selected (Khanmohammadi et al. 2016). During each trial, subjects were asked to stand as still as possible, with their hands alongside the hips, looking straight forward. The level of body balance was determined by the length of the COP path: patch length(cm) and the area of the ellipse 95 percentile Area 95 (cm^2^) (Rombaut et al. 2011; Ruhe et al. 2011).

### Physical fitness measurements

To assess the components of functional fitness the Senior Fitness Test (SFT), a battery of six items, was applied. The SFT items include the following: (1) 30-s chair stand; (2) arm curl; (3) chair sit-and-reach; (4) back scratch; (5) 2-min step; and (6) 8-foot up-and-go. The test was performed in this order with 1 min rest breaks in between components. Before each test, with the exception of the walking test performed only once, the evaluator demonstrated the exercise and the participant carried out an attempt to familiarize herself (Jones et al. 1999).

### Statistical analysis

All measures were compiled in a spreadsheet for the analysis of parallel-group trials and the effects were interpreted using magnitude-based inferences (Hopkins 2006). All data were log-converted to reduce bias arising from error non-uniformity. To improve precision of estimates, mean changes were adjusted to the overall mean baseline blood level of vitamin D3 more and less than 20 ng mL^−1^. Baseline values were expressed in measurement units. Means of the observed and adjusted changes, standard deviations of the observed changes, and adjusted effects (differences in the changes of the means and their certainty limits) were back-transformed to percentages. Magnitudes of the effects were also evaluated with the log-transformed data by standardizing with the means change in women with baseline blood level of vitamin D more than 20 ng mL^−1^ minus adjusted mean change in women with baseline blood level of vitamin D less than 20 ng mL^−1^. Threshold values for difference in means divided by SD of women with baseline blood level of vitamin D3 more than 20 ng mL^−1^ < 0.20; 0.20–0.59; 0.60–1.19; > 1.20, for trivial, small, moderate and large respectively (Hopkins et al. 2009).

## Results

All 27 seniors women completed the study with no adverse events being reported. Baseline descriptive characteristics are presented in Table 1.

**Table 1:** Anthropometric characteristics of participants (n = 27)

	Baseline blood level of vitamin D_3_	Baseline mean ± SD	Observed change mean ± SD (%)
Weight (kg)	Less than 20 ng mL^−1^	67.4 ± 9.5	0 ± 6
	More than 20 ng mL^−1^	67.3 ± 8.4	0 ± 2
BMI (kg m^−2^)	Less than 20 ng mL^−1^	25.4 ± 3.3	1 ± 9
	More than 20 ng mL^−1^	24.9 ± 3.5	4 ± 13
Fat (kg)	Less than 20 ng mL^−1^	24.0 ± 7.3	−2 ± 22
	More than 20 ng mL^−1^	22.7 ± 6.4	−2 ± 5
Fat (%)	Less than 20 ng mL^−1^	34.9 ± 6.8	−1 ± 18
	More than 20 ng mL^−1^	34.1 ± 6.4	−3 ± 6
FFM (kg)	Less than 20 ng mL^−1^	43.4 ± 4.4	0 ± 6
	More than 20 ng mL^−1^	44.1 ± 4.4	2 ± 6
TBW (kg)	Less than 20 ng mL^−1^	31.8 ± 3.2	0 ± 6
	More than 20 ng mL^−1^	32.3 ± 3.2	2 ± 6
SSM (kg)	Less than 20 ng mL^−1^	23.5 ± 2.6	1 ± 7
	More than 20 ng mL^−1^	23.9 ± 2.5	3 ± 6

Values are means (± SD); Observed changes are expressed in percentages

*BMI* body mass index, *Fat* fat mass, *Fat* *%* percentage of body fat, *FFM* free fat mass, *TBW* total body water, *SMM* skeletal muscle mass

There were no significant differences between participants’ body weight, body composition and BMI across the two groups: LVD and MVD and baseline and after intervention. Baseline values of the measured proteins are presented in Table 2. No significant differences were noted in concentrations of myokines and other assessed proteins between groups (LVD and MVD) at the beginning of experiment. The applied NW training program combined with vitamin D supplementation resulted in a slight rise in irisin in the MVD group, whereas in the LVD group the tendency was opposite. The adjusted effect of these changes for irisin was moderate (Table 2).

**Table 2:** The inflammatory markers response induced by 12 weeks of Nordic Walking training

	Baseline blood level of vitamin D_3_	Baseline mean ± SD	Observed change mean ± SD (%)	Adjusted change^a^ mean ± SD (%)	Adjusted effect^b^	
					Mean; CI (%)	Inference^c^
Irisin (ng mL^−1^)	Less than 20 ng mL^−1^	11 ± 3	−23 ± 60	−19 ± 32	26	**Moderate** ↑**
	More than 20 ng mL^−1^	10 ± 3	5 ± 55	3 ± 55	−2 to 63	
HMGB- 1 (ng mL^−1^)	Less than 20 ng mL^−1^	13 ± 13	116 ± 200	56 ± 160	−42	**Moderate** ↓**
	More than 20 ng mL^−1^	27 ± 16	−30 ± 156	−10 ± 132	−71 to 15	
IL6 (pg mL^−1^	Less than 20 ng mL^−1^	1.0 ± 0.4	12 ± 59	15 ± 57	−23	**Moderate** ↓**
	More than 20 ng mL^−1^	0.9 ± 0.3	−10 ± 66	−11 ± 47	−44 to 6	
IL10 (pg mL^−1^)	Less than 20 ng mL^−1^	0.3 ± 0.2	−11 ± 412	1 ± 141	10	Trivial
	More than 20 ng mL^−1^	0.2 ± 0.1	34 ± 440	11 ± 136	−40 to 101	
BDNf (ng mL^−1^)	Less than 20 ng mL^−1^	25 ± 9	−2 ± 114	−12 ± 60	−9	Small
	More than 20 ng mL^−1^	28 ± 9	−25 ± 112	−20 ± 90	−37 to 33	
Valine (µmol L^−1^)	Less than 20 ng mL^−1^	76 ± 20	11 ± 33	8 ± 27	0	Trivial
	More than 20 ng mL^−1^	81 ± 23	6 ± 52	9 ± 36	−16 to 20	
Leucine (µmol L^−1^)	Less than 20 ng mL^−1^	77 ± 22	37 ± 68	37 ± 37	−20	**Moderate** ↓**
	More than 20 ng mL^−1^	80 ± 34	9 ± 61	10 ± 53	−38 to 3	
Isoleucine (µmol L^−1^)	Less than 20 ng mL^−1^	60 ± 21	−26 ± 73	−22 ± 50	15	Small
	More than 20 ng mL^−1^	53 ± 17	−6 ± 53	−10 ± 37	−10 to 46	

CI—90% confidence interval. All data are percentages, with the exception of baseline values expressed in measurement units. Inferences shown in bold are clear at the 98% level of confidence

*HMGB-1* high mobility group box 1; *IL6* interleukin 6; *IL10* interleukin 10; *BDNf* brain-derived neurotrophic factor

^a^Adjusted to overall mean of in baseline blood level of vitamin D3 more and less than 20 ng mL^– 1^

^b^Adjusted mean change in women with baseline blood level of vitamin D3 more than 20 ng mL^−1^ minus adjusted mean change in women with baseline blood level of vitamin D3 less than 20 ng mL^– 1^

^c^Magnitude thresholds (for difference in means divided by SD of women with baseline blood level of vitamin D3 more than 20 ng mL^−1^): < 0.20, trivial; 0.20–0.59, small; 0.60–1.19, moderate; > 1.20, large

↑—Increase; ↓—decrease

*Asterisks* indicate effects clear at the 5% level and likelihood that the true effect is substantial: **—likely

**Fig. 1:**
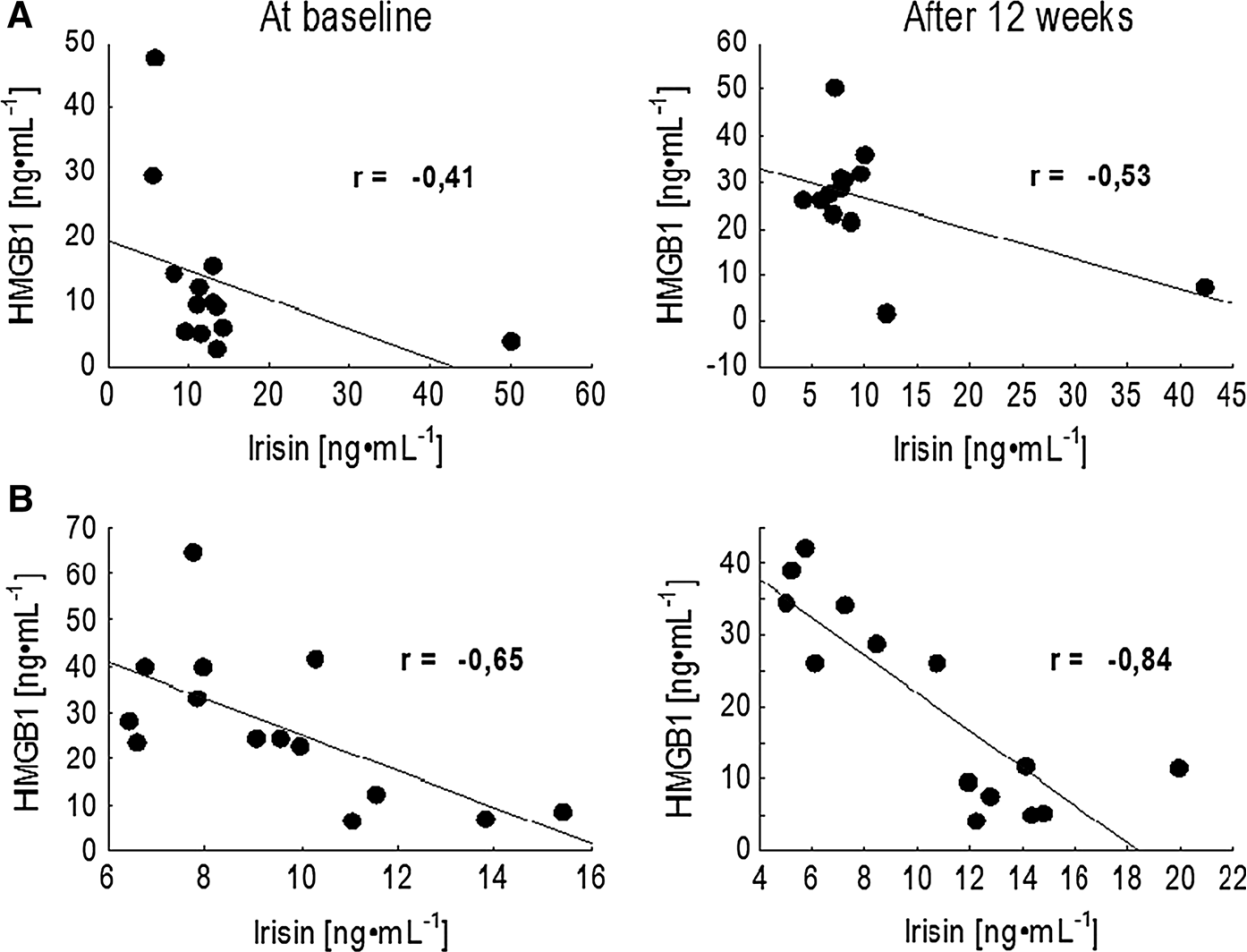
Correlation between irisin and HMGB1 at the baseline and in response to 12 weeks Nordic Walking training in elderly women: **a** group LVD with baseline blood vitamin D level less than 20 ng mL^−1^, **b** group MVD with baseline blood vitamin D level more than 20 ng mL^−1^

At the same time, a significant drop in the pro-inflammatory protein HMGB1 was recorded in the MVD participants. Within the LVD group, the level of HMGB1 increased after the 12 weeks of training. A similar tendency of change was noted in the pleiotropic cytokine IL-6 concentration. The adjusted effect for alternation of IL-6 was also moderate (Table 2). Strikingly, significant negative correlations between irisin and HMGB1 were observed in both groups not only at baseline, but also after the intervention. Still, the relationship between these proteins was much more pronounced in the MVD group and it is worth noting that the NW training enhanced this effect (Fig. 1).

Also in the LVD group the higher the irisin level was, the lower HMGB1 level was recorded. No significant alternations were recorded in the anti-inflammatory cytokine IL-10. Also, changes in the BDNF concentration were insignificantly small.

Among branched amino acids significant changes were only observed in the leucine concentration. Although the applied NW training program combined with vitamin D supplementation caused the leucine level to rise, a comparison of the recorded response between groups and the adjusted effect indicated that the effect of the intervention was 20% lower in the LVD group (Table 2).

The muscle strength analysis did not show any significant changes in knee strength (data not present). Elbow strength measurements are outlined in Table 3.

**Table 3:** Changes in arms strength measurement in response to Nordic Walking training in elderly women

	Baseline blood level of vitamin D_3_	Baseline mean ± SD	Observed change mean ± SD (%)	Adjusted change^a^ mean ± SD (%)	Adjusted effect^b^	
					Mean; CI (%)	Inference^c^
Izokinetic elbow extention 90						
Left peak torque (Nm)	Less than 20 ng mL^−1^	25 ± 7	−7 ± 21	−7 ± 20	5	Small
	More than 20 ng mL^−1^	26 ± 5	−4 ± 31	−2 ± 22	−7 to 19	
Left agon/antag ratio	Less than 20 ng mL^−1^	94 ± 16	9 ± 19	9 ± 17	−5	Small
	More than 20 ng mL^−1^	96 ± 19	2 ± 36	3 ± 19	−16 to 6	
Right peak torque (Nm)	Less than 20 ng mL^−1^	23 ± 4	5 ± 29	1 ± 19	−3	Trivial
	More than 20 ng mL^−1^	25 ± 4	−4 ± 25	−2 ± 25	−15 to 11	
Right Agon/antag ratio	Less than 20 ng mL^−1^	102 ± 20	1 ± 30	1 ± 14	3	Trivial
	More than 20 ng mL^−1^	101 ± 15	4 ± 18	4 ± 17	−6 to 14	
Izokinetic elbow Flexion 90						
Left peak torque (Nm)	Less than 20 ng mL^−1^	25 ± 6	−1 ± 22	0 ± 17	−3	Trivial
	More than 20 ng mL^−1^	24 ± 5	−2 ± 25	−3 ± 16	−12 to 8	
Right peak torque (Nm)	Less than 20 ng mL^−1^	23 ± 6	6 ± 31	3 ± 22	0	Trivial
	More than 20 ng mL^−1^	25 ± 3	0 ± 25	2 ± 24	−13 to 15	
Izometric elbow away						
Left peak torque (Nm)	Less than 20 ng mL^−1^	26 ± 7	18 ± 30	14 ± 24	−3	Trivial
	More than 20 ng mL^−1^	28 ± 6	7 ± 43	11 ± 28	−17 to 14	
Left Agon/antag ratio	Less than 20 ng mL^−1^	124 ± 25	−4 ± 40	−2 ± 29	9	Small
	More than 20 ng mL^−1^	115 ± 12	8 ± 20	6 ± 20	−8 to 28	
Right peak torque (Nm)	Less than 20 ng mL^−1^	29 ± 7	9 ± 22	9 ± 23	−8	Small
	More than 20 ng mL^−1^	29 ± 5	0 ± 24	0 ± 24	−20 to 7	
Izometric elbow toward						
Left peak torque (Nm)	Less than 20 ng mL^−1^	34 ± 7	3 ± 29	4 ± 20	2	Small
	More than 20 ng mL^−1^	33 ± 6	6 ± 13	6 ± 12	−8 to 13	
Left agon/antag ratio	Less than 20 ng mL^−1^	119 ± 23	−11 ± 20	−11 ± 21	20	**Moderate ↑** ^***^
	More than 20 ng mL^−1^	117 ± 26	8 ± 27	7 ± 19	6 to 36	
Right peak torque (Nm)	Less than 20 ng mL^−1^	31 ± 8	12 ± 28	9 ± 13	4	**Trivial ↑** ^*^
	More than 20 ng mL^−1^	33 ± 8	11 ± 18	13 ± 12	−4 to 13	

CI—90% confidence interval. All data are percentages, with the exception of baseline values expressed in measurement units. Inferences shown in bold are clear at the 98% level of confidence

^a^Adjusted to overall mean of in baseline blood level of vitamin D3 more and less than 20 ng mL – 1

^b^Adjusted mean change in women with baseline blood level of vitamin D3 more than 20 ng mL^−1^ minus adjusted mean change in women with baseline blood level of vitamin D3 less than 20 ng mL^−1^

^c^Magnitude thresholds (for difference in means divided by SD of women with baseline blood level of vitamin D3 more than 20 ng mL^−1^): < 0.20, trivial; 0.20–0.59, small; 0.60–1.19, moderate; > 1.20, large

↑—Increase; ↓—decrease

*Asterisks* indicate effects clear at the 5% level and likelihood that the true effect is substantial: *—possible, ***—very likely

The intervention induced significant changes in isometric elbow toward. There were moderate very likely increase adjusted effect on left Agon/antag ratio and trivial possible adjusted effect on right peak torque. Changes in isokinetic elbow strength were small, trivial and unclear. Together with changes in arm muscle strength, shifts in body balance were noted. Characteristics of body balance indicators are presented in Table 4.

**Table 4:** Alteration of body balance in response to the intervention of Nordic Walking training

	Baseline blood level of vitamin D_3_	Baseline mean ± SD	Observed change mean ± SD (%)	Adjusted change^a^ mean ± SD (%)	Adjusted effect^b^	
					Mean; CI (%)	Inference^c^
EO min PathLgth (cm s^−1^)	Less than 20 ng mL^−1^	37 ± 5	8 ± 23	5 ± 19	−6	Small
	More than 20 ng mL^−1^	39 ± 5	−2 ± 16	−2 ± 17	−16 to 5	
EO min Area95 (cm s^−1^)	Less than 20 ng mL^−1^	0.7 ± 0.3	52 ± 152	72 ± 81	−43	**Moderate** ↓^***^
	More than 20 ng mL^−1^	0.6 ± 0.4	3 ± 108	−3 ± 70	−61 to −17	
EC minPathLgth (cm s^−1^)	Less than 20 ng mL^−1^	48 ± 8	4 ± 27	5 ± 14	−1	Trivial
	More than 20 ng mL^−1^	46 ± 8	5 ± 19	4 ± 20	−13 to 11	
EC min Area95 (cm s^−1^)	Less than 20 ng mL^−1^	1.1 ± 0.6	33 ± 94	58 ± 54	4	Trivial
	More than 20 ng mL^−1^	1.0 ± 1.3	81 ± 123	64 ± 98	−32 to 57	
SR min PathLgth (cm s^−1^)	Less than 20 ng mL^−1^	121 ± 28	−6 ± 14	−6 ± 14	4	Trivial
	More than 20 ng mL^−1^	134 ± 41	−4 ± 21	−3 ± 19	−7 to 16	
SR min Area95 (cm s^−1^)	Less than 20 ng mL^−1^	5.7 ± 1.5	−11 ± 44	−10 ± 43	6	Trivial
	More than 20 ng mL^−1^	5.4 ± 2.3	−4 ± 50	−5 ± 51	−19 to 38	
SL min PathLgth (cm s^−1^)	Less than 20 ng mL^−1^	120 ± 22	−4 ± 16	−6 ± 17	−1	Trivial
	More than 20 ng mL^−1^	143 ± 60	−6 ± 36	−6 ± 29	−15 to 16	
SL min Area95 (cm s^−1^)	Less than 20 ng mL^−1^	4.9 ± 1.2	−13 ± 48	−13 ± 51	−7	Trivial
	More than 20 ng mL^−1^	6.0 ± 3.5	−23 ± 80	−19 ± 58	−32 to 27	

CI—90% confidence interval. Measurements position: *EO* eyes open, *EC* eyes closed, *SR* single right, *SL* single left, *TR* balance position with the right foot forward, *TL* balance position with the left foot forward. All data are percentages, with the exception of baseline values expressed in measurement units. Inferences shown in bold are clear at the 98% level of confidence

^a^Adjusted to overall mean of in baseline blood level of vitamin D3 more and less than 20 ng mL^−1^

^b^Adjusted mean change in women with baseline blood level of vitamin D3 more than 20 ng mL^−1^ minus adjusted mean change in women with baseline blood level of vitamin D3 less than 20 ng mL^−1^

^c^Magnitude thresholds (for difference in means divided by SD of women with baseline blood level of vitamin D3 more than 20 ng mL^−1^): < 0.20, trivial; 0.20–0.59, small; 0.60–1.19, moderate; > 1.20, large

↑—Increase; ↓—decrease

*Asterisks* indicate effects clear at the 5% level and likelihood that the true effect is substantial: ***—very likely

Obtained data pointed that very likely moderate decrease adjusted effect, was noted in Eyes Open min Area95, which indicated on improvement of the body balance among women with higher initial vitamin D concentration. Changes and adjusted effect on components of functional fitness are no significance (Table 5), excluding small increase adjusted effect in chair stand test (possible, 98%).

**Table 5:** Changes of functional fitness components in the senior fitness test (SFT) after 12 weeks of Nordic Walking training

	Baseline blood level of vitamin D_3_	Baseline mean ± SD	Observed change mean ± SD (%)	Adjusted change^a^ mean ± SD (%)	Adjusted effect^b^	
					Mean; CI (%)	Inference^c^
Chair stand (no. of stands)	Less than 20 ng mL^−1^	21 ± 3	0 ± 21	0 ± 22	8	**Small** ↑^*^
	More than 20 ng mL^−1^	21 ± 4	10 ± 15	8 ± 9	−3 to 20	
Arm curl (no. of reps)	Less than 20 ng mL^−1^	25 ± 5	− 8 ± 87	−8 ± 93	22	Moderate
	More than 20 ng mL^−1^	27 ± 6	9 ± 16	12 ± 13	−15 to 74	
2000 m walk test (m)	Less than 20 ng mL^−1^	1117 ± 121	−3 ± 7	−2 ± 6	−2	Trivial
	More than 20 ng mL^−1^	1032 ± 76	−2 ± 9	−3 ± 9	−7 to 4	
Chair sit-and-reach (cm ±)	Less than 20 ng mL^−1^	7 ± 8	−14 ± 68	−13 ± 74	39	Small
	More than 20 ng mL^−1^	11 ± 9	8 ± 143	20 ± 111	−17 to 130	
Back scratch (cm ±)	Less than 20 ng mL^−1^	0 ± 6	24 ± 94	10 ± 62	−26	Small
	More than 20 ng mL^−1^	1 ± 5	−17 ± 106	−18 ± 117	−59 to 34	
8-Foot up-and-go (s)	Less than 20 ng mL^−1^	4.82 ± 0.5	−10 ± 8	−10 ± 9	−2	Trivial
	More than 20 ng mL^−1^	4.62 ± 0.6	−11 ± 17	−12 ± 10	−8 to 4	

CI—90% confidence interval. All data are percentages, with the exception of baseline values expressed in measurement units. Inferences shown in bold are clear at the 98% level of confidence

^a^Adjusted to overall mean of in baseline blood level of vitamin D3 more and less than 20 ng mL^−1^

^b^Adjusted mean change in women with baseline blood level of vitamin D3 more than 20 ng mL^−1^ minus adjusted mean change in women with baseline blood level of vitamin D3 less than 20 ng mL^−1^

^c^Magnitude thresholds (for difference in means divided by SD of women with baseline blood level of vitamin D3 more than 20 ng mL^−1^): < 0.20, trivial; 0.20–0.59, small; 0.60–1.19, moderate; > 1.20, large

↑—Increase; ↓—decrease

*Asterisks* indicate effects clear at the 5% level and likelihood that the true effect is substantial: *—possible

**Fig. 2:**
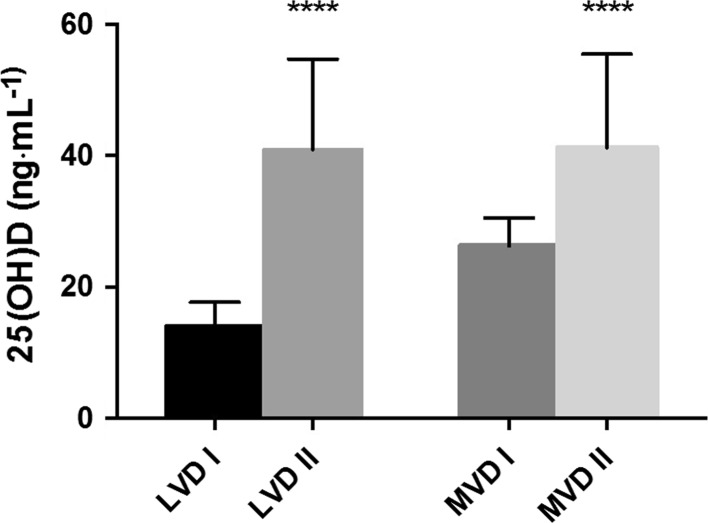
Changes in concentration of vitamin D at the baseline (I) and after whole intervention (II) in booth groups: LVD group with baseline blood vitamin D level less than 20 ng mL^−1,^ MVD group with baseline blood vitamin D level more than 20 ng mL^−1^

Most women taking part in the experiment were experiencing significant vitamin D deficiency. Applied supplementation significantly improved concentrations of vitamin D in both groups with the final concentration equal 40.98 ± 14.0 ng mL^−1^ in LVD and 41.25 ± 26.3 ng mL^−1^ in MVD group respectively (Fig. 2).

### Effect size is large for booth groups

The doses of vitamin D supplementation did not significantly modify response to training. However, the number of subjects in subgroups was limited and in should be in further investigation to enhance. Looking across the whole group of participants, between women supplemented with 800 IU and 4000 IU more significant changes in muscle strength arms were recorded among women supplemented with higher doses of vitamin D.

## Discussion

This study revealed that 12 weeks of Nordic Walking training in elderly women lead to a decrease the autophagy-inducing stress protein HMGB1. This is the first study which demonstrates the effect of exercise on changes HMGB1 in elderly people. It has been demonstrated that during the development of colorectal cancer, serum HMGB1 increased, thus changes in opposite direction induced by NW training should be considered as a positive one. Moreover, the presence of HMGB1 receptors TLR4 and RAGE were identified in skeletal muscle and binding this protein by receptors is known to stimulate muscle protein degradation (Luo et al. 2014). Only few studies revealed the reduction HMGB1 concentration in response to exercise. Giallauria et al. demonstrated that one year, moderate intensity training applied also in breast cancer survivors significantly decreased HMGB1 concentration (Giallauria et al. 2014). Inflammation in elderly people is a strong predictor of both disability and mortality, even in the absence of a clinical disease (Kragstrup et al. 2011). Most importantly, chronic inflammation contributes to skeletal muscle mass and function loss, leading to an earlier onset of disability. Mechanisms underpinning this loss are complex, but may include age-related changes in the immune function, previously described as “inflamm-aging” (Franceschi et al. 2000). The applied NW training program induced a drop in HMGB1 with the effect size much more pronounced among elderly subjects characterized by an elevated concentration of vitamin D at the baseline. At the same time, a significant increase in arms muscle strength was noted. Contrary to previous observations (Ossowski et al. 2016), in the current study NW had no effect on leg muscle strength (data not presented). Similarly, as noted before (Kortas et al. 2015), NW training resulted in a significant decrease in pro-inflammatory marker interleukin-6. IL-6 can be considered both a pro- and an anti-inflammatory protein. Significant shifts in IL-6 in response to exercise, its intensity and duration, working muscle mass and individual’s endurance capacity was reported in previous papers (Febbraio and Pedersen 2005; Petersen and Pedersen 2005). In the current study, the resting level of IL-6 was measured at baseline and three days after the whole training program; thus, we assumed that the IL-6 concentration after 12 weeks is a marker of inflammation. However, this is only a speculation as it is difficult to establish the source of IL-6; it would require applying advanced methods such as muscle biopsies and RT-PCR (Keller et al. 2001).

Diminished concentrations of pro-inflammatory IL-6 and HMGB1 were accompanied by the rise of myokine-irisin. Moreover, a significant inverse correlation between this myokine and HMGB1 was observed at baseline as well as after the NW training program. It is worth highlighting that the drop was more pronounced in the group of women with higher concentration of vitamin D. Although exercise is known for its ant-inflammatory action (Petersen and Pedersen 2005), in many cases different factors may limit adaptive changes. Vitamin D deficiency recorded in the current study cannot be excluded as one of these factors modifying exercise-induced changes.

Based on literature data we assumed that NW exercise would cause an increase in irisin. This rise in irisin was expected to stimulate an increase in the BDNF concertation (Wrann et al. 2013; Zsuga et al. 2016). Nonetheless, our initial hypothesis was only partially confirmed by the obtained findings. A significant rise in irisin was noted in both groups. Still the effect of training was higher in the MVD group, although in both groups no significant shifts in the BDNF concentration were observed. Previously, low BDNF was noted among aging, obese, Alzheimer patients and dementia subjects (Knaepen et al. 2010). It is also known that together with muscle mass impairment, age-related cognitive functions decline (Wongrakpanich et al. 2016). The majority of the studies concluded that physical activity in later life confers a protective effect on cognition in elderly subjects (Carvalho et al. 2014). Zoladz et al. also revealed that in response to a moderate-intensity 8-week interval training program, BDNF concentration grew significantly among Parkinson patients (Zoladz et al. 2014). Although the BDNF concentration did not alter in response to the NW training, improvement of body balance was achieved. Balance control depends on a number of central and peripheral factors (Baudry 2016). Augmentation of BDNF uptake provides an interpretation of the obtained body balance results as it induces improvement of the nervous system function. Further investigation, however, would be required to confirm this interpretation.

An increase in arm muscle strength and body balance improvement were accompanied by changes in the leucine concentration. We assumed that blood levels of leucine demonstrate the free pool of this amino acid that remains after cellular uptake. Anabolic resistance of the muscle protein synthesis is commonly observed with aging (Burd et al. 2013). Leucine has been described as an important essential amino acid and a nutrient signal that activates mTOR leading to increased muscle protein synthesis (Ananieva et al. 2016). Some study provided the evidence, that vitamin D and leucine-enriched whey protein supplement resulted in improvements of muscle mass and lower-extremity function among sarcopenic older adults (Bauer et al. 2015). In our experiment, significant changes in the leucine concentration were noted. Still, the size of changes was higher in the MVD group. It is worth to note, that vitamin D was proposed to be a factor influencing insulin sensitivity and its supplementation was recommended, to prevent diabetes (Van Belle et al. 2013). Thus, in the MVD group vitamin D may have enhanced the anabolic response causing the better uptake of leucine. Another explanation of the decrease observed in the serum leucine concentration may be connected with the drop of HMGB1, which is known to stimulate amino acids release from skeletal muscle (Luo et al. 2014).

The doses of vitamin D had no significant impact on the obtained results. Numbers of participants in supplementation subgroups of the LVD and MVD groups were limited due to the attendance requirement of minimum 90% training units. Consequently, it was decided not to analyze the results this way. Since reports exist on a range of vitamin D supplementation doses (Rosendahl-Riise et al. 2017), research should be continued to fully recognize effects of that treatment.

In conclusion, our study confirms that physical exercise is associated to reduced negative age-related changes. This data support the notion that vitamin D is one of the factors that mediate the relationship between exercise, inflammation and muscle function. Pro-healthy effects of the NW training is manifested by a decreased concertation of pro-inflammatory proteins such HMGB1 and IL 6 and increase myokine having role of metabolic regulation such irisin. In addition NW could improves leucine uptake and muscles protein synthesis, however further investigation is needed to explain the exact mechanism observed changes. Thus this form of exercise can be consider as the effective training, which can prevent muscle sarcopenia.
